# HIV and Mediterranean Zoonoses: A Review of the Literature

**DOI:** 10.3390/idr14050075

**Published:** 2022-09-16

**Authors:** Ylenia Russotto, Cristina Micali, Giovanni Francesco Pellicanò, Giuseppe Nunnari, Emmanuele Venanzi Rullo

**Affiliations:** 1 Unit of Infectious Diseases, Department of Clinical and Experimental Medicine, University of Messina, 98124 Messina, Italy; 2 Department of Human Pathology of the Adult and the Developmental Age “G. Barresi”, University of Messina, 98124 Messina, Italy

**Keywords:** HIV, zoonoses, brucellosis, borrelia, monkeypox, campylobacteriosis, rickettsia, listeria, salmonellosis, echinococcosis

## Abstract

A zoonosis is an infectious disease that has jumped from a non-human animal to humans. Some zoonoses are very common in the Mediterranean area and endemic in specific regions, so they represent an important problem for public health. Human Immunodeficiency Virus (HIV) is a virus that has originated as a zoonosis and is now diffused globally, with the most significant numbers of infected people among the infectious diseases. Since the introduction of antiretroviral therapy (ART), the history for people living with HIV (PLWH) has changed drastically, and many diseases are now no different in epidemiology and prognosis as they are in not-HIV-infected people. Still, the underlying inflammatory state that is correlated with HIV and other alterations related to the infection itself can be a risk factor when infected with other bacteria, parasites or viruses. We reviewed the literature for infection by the most common Mediterranean zoonoses, such as *Campylobacter*, *Salmonella*, *Brucella*, *Rickettsia*, *Borrelia*, *Listeria* and *Echinococcus*, and a possible correlation with HIV. We included *Monkeypox*, since the outbreak of cases is becoming a concern lately. We found that HIV may be related with alterations of the microbiome, as for campylobacteriosis, and that there are some zoonoses with a significant prevalence in PLWH, as for salmonellosis.

## 1. Introduction

A zoonosis, according to the World Health Organization (WHO), is defined as an infectious disease that has jumped from a non-human animal to a human and, therefore, a disease that is transmissible from animal to humans. Some infectious diseases began as a zoonosis and then developed as human-to-human transmitted diseases; among these there is human immunodeficiency virus (HIV), which developed as a result of multiple cross-species transmissions from SIV (simian immunodeficiency virus), naturally infecting African primates [1].

The definition provided by WHO requires a bit of narrowing to differentiate the various kinds of zoonoses. Zoonoses commonly indicate those diseases caused by pathogens that can replicate in the animal host, not including diseases caused by toxins or venoms. Regarding the transmissibility, zoonoses are considered those diseases that can be transmitted from animal to humans via an insect vector or via direct contact with the animal or its products [2]. Zoonotic infections can be transmitted to humans through many routes, the most common being animal bites and scratches, contaminated animal food products, through work exposure such as in jobs such as veterinarians or farmers, arthropods as vectors or contaminated soil and watercourses.

Zoonoses can be caused by many pathogens, such as viruses, bacteria, parasites and prions.

Globalization has guaranteed an easier route for some pathogens to transmit and the continuous flow of people traveling the world has exposed a large number of humans to many different cultural and alimentary customs, previously restricted to a small area. A growing number of microbes have jumped from their usual reservoir and have become able to infect humans [3,4,5].

The most recent example of this natural process, defined as “spillover”, is the jump of SARS-CoV-2 from mammals to humans in 2019, which led to a pandemic [6].

Among all the animals causing known and emerging zoonoses, bats have been under observation as one of the main reservoirs for viruses. Even though it is impossible to calculate the risk of a specific virus to spill over, it has been observed that RNA viruses are most likely to jump between species, as the *Ebola* epidemic and SARS-CoV-2 pandemic demonstrate [7].

Due to SARS-CoV-2 disruption, many infectious disease diagnoses, such as tuberculosis, have been delayed [8]. Among these, HIV testing and thus diagnosis have slowed down and a spread of new infections is being observed globally, with many tardive diagnoses, formulated after casual discovery of cancers or other acquired immunodeficiency syndrome (AIDS)-defining illnesses [9]. Meanwhile, the number of zoonotic diseases in many areas is still a public health problem.

Zoonotic infections, such as brucellosis, echinococcosis, leishmaniasis, rabies and zoonotic salmonellosis are very common in the Mediterranean area. In 1978, the Mediterranean Zoonoses Control Center (MZCC) was established. The main reason behind the great spread of zoonoses in the Mediterranean area is the favorable climate for mosquitos and other kinds of arthropods. Therefore, the Mediterranean area can be defined as a hotspot for the development and transmission of most common zoonotic infections. The control over the environment and the local animal species has limited the spread of these kinds of diseases, while the Middle East and the African regions have become a matter of concern for emerging infectious diseases. However, the diseases transmitted by animals, such as brucellosis, rickettsiosis, and leishmaniasis, are still a strong component of the infectious diseases diagnoses in the Mediterranean area [10].

The immune-deficiency due to HIV infection is a major risk factor, not only for the development of cancers [11,12,13,14,15,16,17,18,19,20], but also for other infectious diseases defined as “opportunistic”. Some of them are characteristic of the AIDS stage of HIV infection and well known from the pre-antiretroviral therapy (ART) period; those are what we call AIDS-defining illnesses.

Meanwhile, infections in people living with HIV (PLWH) by other bacteria, not considered characteristic of HIV infection, are not so rare [21]. The presence of an HIV reservoir in T-cells of PLWH under ART [22] may lead to a different response from the immune system to common zoonoses, especially the ones that usually elicit a T-cell based response. The ART itself, by affecting the underlying inflammation by various mechanisms [23], may be related to a different kind of response of PLWH to some zoonoses.

We decided to analyze the more neglected zoonoses, typical of the Mediterranean area, in relation to HIV.

## 2. Epidemiology

According to the OneHealth report from the European Centre for Disease Prevention and Control (ECDC) and the European Food Safety Authority (EFSA), in 2020, campylobacteriosis has been confirmed to be the most frequent zoonosis in the European Union region, accounting for 60% of zoonotic disease diagnoses with 120,946 cases (Table 1). The following most prevalent zoonoses are salmonellosis, yersioniosis, infections by shiga toxin-producing *Escherichia coli* (STEC) and listeriosis. In the same year, there were 641 cases of tularemia, and also *Coxiella burnetii* infection, even if sporadic outbreaks associated with contaminated waters and mosquito’s bites were reported [24]. Echinococcosis covered for a grand total of 488 cases in Europe in 2020, and cystic echinococcosis has an annual incidence from <1 to 200 per 100,00 in endemic areas, such as western China, Central Asia, South America, Mediterranean countries and eastern Africa [25]. In 2020, brucellosis was accountable for 128 new diagnoses in the European regions. According to the OneHealth report and ECDC data between 2016 and 2020, a significant reduction in the incidence of brucellosis diagnoses has been observed in European countries, even if it remains endemic in specific areas including the Mediterranean basin, the Middle East, the Indian subcontinent, Latin America and some African countries [26].

Regarding HIV infections, according to World Health Organization (WHO) data, there were an estimated 37.7 million PLWH at the end of 2020, with 1.5 million new diagnoses in 2020, and 104,765 in 46 out of 53 of the European regions that belong to the WHO Europe.

## 3. Campylobacteriosis

*Campylobacter* is a genus of bacteria of the family Campylobacteraceae and, to date, comprehends a total of 32 species and 9 subspecies. The most common is *C. jejuni*, and to a lesser extent, *C. coli*.

In a systematic review published in 2021 on campylobacteriosis in the sub-Saharan African regions by Hlashwayo et al. [27], campylobacteriosis was found to have no difference in prevalence in HIV-infected and non-HIV-infected patients, while a higher prevalence was found in the HIV group with diarrhea compared to the ones with no diarrhea. This data confirmed the findings of a previous study on gastroenteritis in 235 MSM in 2017–2018 by Newman et al. [28], with 148 of them being seropositive and with no differences in *Campylobacter* detection between the HIV and non-HIV groups.

Infection by *Campylobacter* occurs mostly by injection of *Campylobacter*-contaminated raw or undercooked meat, raw milk, tap water and various chicken-containing dishes. The motility of the bacterium is granted by the flagella, which are fundamental for colonization. Adhesion to the intestinal epithelium is mediated by some proteins present on the *Campylobacter* surface, such as CapA or the protein of Campylobacter adhesion, and the cell damage is brought about by the production of cytotoxins [29]. Campylobacteriosis usually involves a self-limiting gastroenteritis, clinically characterized by fever and vomit that may last from one to three days, abdominal pain and diarrhea that may last up to two weeks [30,31].

In many studies, prior to the introduction of ART, a more severe campylobacteriosis is described, demonstrating that the antiretroviral therapy has impacted infections by *Campylobacter* in PLWH in many ways [32].

In a study by Larsen et al. (2011) [33], it was found that the incidence of *Campylobacter*-related diseases in PLWH in Denmark has decreased since the introduction of the ART; in the same study, however, an increase in campylobacteriosis in men who have sex with men (MSM) was also observed.

In a study conducted in Ghana by Forson et al. [34], where the *Campylobacter*-associated gastroenteritis in hospitalized PLWH was evaluated, a high prevalence of *C. coli* was found in the population, with also a significant percentage of resistance genes. Other evidence in the literature report *Campylobacter* to be the most common pathogen associated with diarrhea in PLWH as well [35,36,37], such as in a study conducted by Samie et al. [38], in which *Campylobacter* was proved to be the most common pathogen associated with diarrhea in HIV-infected individuals with a percentage of 22.8%. Similar results were obtained in a study of 215 HIV patients in Ethiopia, where *Campylobacter* was confirmed to be the most common pathogen causing gastroenteritis (6.04%) [39], and in another study with a prevalence of 4.4% [40].

While *Campylobacter* is usually associated with gastrointestinal symptoms, generally self-limiting, there are cases of Campylobacter-related bacteremia reported in the literature. In 1998, the relation between *Campylobacter* bacteremia in 21 PLWH was analyzed by Tee w et al. [41], and the disease was found to be significantly more severe than in non-immunocompromised people, with a mortality rate of 33%. In a study by Fernandez-Cruz et al. [42], *Campylobacter* bacteremia was found to be more severe in HIV-infected patients compared to non-HIV-infected (33% vs. 10%), although it has to be said that the higher prevalence of *Campylobacter* bacteremia in HIV-infected people was chronologically located in the pre-ART era. Particular cases include the one of a cellulitis consequent to *Campylobacter* bacteremia in a HIV patient, which recovered after antibiotic therapy [43], and a case of bacteremia from *Campylobacter jejuni* in an HIV-infected patient at the AIDS stage [44].

The increasing resistance of *Campylobacter* to specific classes of antibiotics, however, has raised concern for this normally self-limiting zoonosis. Some genes have been reported to be associated with aminoglycosides and macrolides resistance, and the efflux pump CmeABC variant is the culprit for increasingly high doses of fluoroquinolones resistance [45]. The emergence of erm(B), instead, is related to high-level macrolide resistance [46].

*Campylobacter* levels are often altered in the gut microbiota in many different diseases [47,48].

In a study conducted in Nigeria by Nowak et al. [49], three populations of MSM were compared: the HIV uninfected, the untreated seropositive people, and the HIV-infected who were receiving ART. Notably there were no differences in *Campylobacter* levels in the microbiome in the first two groups; meanwhile, in PLWH under ART, a significant increase in *Campylobacter* population was found in rectal microbiota.

Significantly, a higher prevalence of *Campylobacter* in PLWH under ART was already proved by a prior study published in 2014 by Li Y et al. [50] in another anatomical region and in different human fluids: the concentration of *Campylobacter* was higher in the saliva of HIV-infected patients who were taking ART.

Meanwhile, in a study by Wells et al. [51] conducted in 50 female patients with HIV and high-risk but without HIV infection, there was no significant difference in anal microbiome concerning the presence of *Campylobacter*. In an analysis conducted on the microbiomes of 383 MSM by Cook et al. [52], comparing HIV-infected with undetectable titer, non-HIV and viremic HIV, *Campylobacter* was found to be decreased in the microbiome of HIV-infected suppressed patients, while it was instead increased in viremic HIV-infected. Similar results were given in a study on the lingual microbiome in untreated HIV people by Dang et al. [53].

Peculiar cases with atypical clinical presentation are reported by Rajendran et al. [54], describing erythematous plaques by *Campylobacter* in a seropositive patient, aortitis by *C. fetus* in an HIV-infected man [55], spondylodiscitis caused by *C. fetus* [56] and persistent diarrhea in an HIV + person with the first isolation of *C. infans* [57].

## 4. Salmonellosis

*Salmonella* is a Gram-negative rods genus belonging to the Enterobacteriaceae family. The most known serotypes belonging to Salmonella spp. are *S. typhi* and *S.* *paratyphi*, which cause enteric fever. Less known, but still responsible for over 1 million infections per year in the US, is the non-*typhi Salmonella* [58].

Salmonellosis is usually contracted through the consumption of contaminated food, mostly eggs, meat, poultry and milk that have not been properly cooked. In 2022, a red flag was put on peanut butter for the increasing number of cases of Salmonellosis. Transmission person-to-person is also possible through the fecal-oral route, and humans can contract Salmonellosis also via contact with domestic or not domestic pets.

Salmonellosis is usually characterized by the onset of fever, abdominal pain, diarrhea, nausea and vomiting. The symptoms usually occur 6–72 h after ingestion of *Salmonella* and can last up to 7 days. They are usually mild and self-limiting, even if in some more fragile patients they can be more severe. Other than the most common manifestations, there have been particular cases of *Salmonella*-related liver abscesses in HIV-infected patients [59,60,61], *Salmonella* meningitides [62,63] and thyroiditis due to *Salmonella* in PLWH [64].

Despite being usually self-limiting in immunocompetent people, it has been demonstrated that PLWH are at major risk of developing bloodstream infection, so much so that recurrent salmonella septicemias have been recognized as an AIDS-defining illness. In particular, those with invasive infections by non-*typhi Salmonella* (iNTS) are the ones at risk of a more severe prognosis [65], especially PLWH not on ART regimen [66]. This is confirmed by the analysis conducted on PLWH and the risk of recurrent non-typhoid salmonellosis by Hung CC et al. [67], which appeared to be reduced after the introduction of ART [68].

In particular, iNTS are a prominent issue in severely immunocompromised HIV-infected people (commonly identified with CD4+ count < 200 cell/uL) in African regions [69,70,71,72,73,74,75,76], often recognized as the cause of a severe bacteremia [77,78]. The severity of the iNTS has caused an epidemic, mostly because of the antibiotic resistance that has raised concern in recent years. In particular, some specific variants of *S. typhimurium* have developed mechanisms of multidrug resistance as a phase 2 flagellum [79]; meanwhile, *S. typhi* seems to have maintained sensibility to commonly used antibiotics [80,81,82]. In a study by Crump et al., HIV seemed to even have a protective role against typhoid fever [83].

The severity of iNTS in PLWH may be due to an impaired immune response to the infection, as suggested in a study by MacLennan et al. [84]; specifically, it was reported that an excess of antibodies against *Salmonella* lipopolysaccharide (LPS) is associated with a reduction in *Salmonella* killing. Other reasons behind the severity of iNTS in PLWH may be found in the dysregulation of proinflammatory cytokine release, such as TNF alpha, IL-10 and IL-12 [85], the attenuation of transcription factor nuclear κB (NFκB)-mediated inflammation [86], the defective activity of monocytes phagocytosis [87] and the apparent enhanced capacity of a specific sequence type 313 (ST313) of *S. typhimurium* of surviving in HIV-infected macrophages and their defective phagocytic ability [88,89].

An interesting study by Dandekar et al. [90] suggests that the depletion of the subset Th17 of CD4 T-helper cells in the gut impairs the immune response in the mucosa, leading to translocation of bacteria such as *S. typhimurium* and its consequent invasive infection.

Introduction of ART has modified radically the history of salmonellosis in HIV-infected people.

## 5. Listeriosis

*Listeria monocytogenes* is an anaerobic bacterium that can contaminate many different kinds of food, such as milk, meat and vegetables. It has the unique capability to infect the fetoplacental system and this makes listeriosis a not so rare disease among newborns and pregnant women. A concerning outbreak of listeriosis has been reported in South Africa by Thomas et al. [91], probably related to polony, a processed meat; in 38% of the pregnancy associated cases, the patients were seropositive, as well as in 46% of the non-pregnancy associated cases.

*Listeria* has various virulence factors, such as Internalin A and B, which can facilitate the cell’s invasion. Bacteremia and neurolisteriosis, in particular meningitidis, are the most common expression of *Listeria* infection [92].

Since the discovery of HIV and the opportunistic AIDS-related infections in late 1980s, there have been several cases of listeriosis in PLWH [93,94], and the most common manifestation was meningitis [95,96].

Despite remaining infrequent, as opportunistic infection, few cases of Listeria-related meningitis have been reported in PLWH, even after the introduction of ART [97,98,99,100]; sometimes listeriosis led to further examinations and diagnosis of HIV [101,102]. Few cases of bacteremia have been reported as well [103,104].

In a study conducted on pregnant women in Brazil by Freitag et al. [105], seroprevalence for *Listeria* was found to be associated with HIV; on the other hand, no association with HIV and prevalence of *Listeria* in feces was found in another study by the same authors [106].

Atypical manifestations of listeriosis in PLWH include aortic aneurysm [107], peritonitis [108], prostatitis [109] and a case of cholecystitis and sepsis [110].

## 6. Echinococcosis

*Echinococcus* is a globally distributed parasite. The main species of *Echinococcus* are *E. granulosus*, which cause cystic echinococcosis (CE), diffused throughout Africa, Europe, Asia, the Middle East, Central and South America, and *E. multilocularis*, which causes alveolar echinococcosis (AE), prevalent in the northern latitudes of Europe, Asia, and North America. According to ECDC reports, in 2020, 529 confirmed echinococcosis cases were reported in the European Union. Among these, 243 cases were reported as *Echinococcus granulosus*, 114 as *Echinococcus multilocularis*, and 172 were reported as unknown species. The European Register of CE (ERCE) is a platform originated in 2014, with 15 countries (7 not European) affiliated to it, with the purpose of showing that CE is a relevant issue for public health. Since its foundation, 3386 cysts have been recorded at first registration [111].

The diffusion of echinococcosis is related to dogs, in which the cysts develop into adult tapeworms. Tapeworm’s eggs are diffused in the ground through the dog’s feces and animals can get infected by ingesting them. Humans get the infection usually by consumption of infected water, soil or food.

CE usually remains asymptomatic until the hydatid cysts grow large enough to provoke nausea, discomfort and pain. It may take several years to become symptomatic. The cysts can usually be found in the liver and lungs. CE is associated with polyfunctional T-cell subsets (as IL-2+TNF-α+Th2+ triple-positive and TNF-α+Th2+ double-positive T-cells) related to cyst biological activity [112].

In AE, *E. multilocularis* do not fully develop as cysts; rather, it generally causes vesicles in the liver and may diffuse to other organs.

Notably, in a study by Wahlers et al. [113,114], it was hypothesized that HIV and/or tuberculosis infection may orient the host’s immune response towards Th-2, and Th-2 response was demonstrated to have an association with active CE and resistance to albendazole treatment.

However, to date, there is no strong evidence of a correlation between HIV and echinococcosis, mostly being cases of sporadic infection by *Echinococcus* in PLWH.

There are no studies concerning echinococcosis in PLWH in the Mediterranean area. However, in a study conducted in Mozambique, the prevalence of parasitosis among PLWH has been evaluated: echinococcosis accounted for 17.3% of the overall prevalence [115]. More recently, the prevalence of echinococcosis in immunocompromised patients has been evaluated in a review by Ghasemirad et al. [116], and HIV resulted in the most common condition in CE.

The vast majority of cases reported in the literature concerning infection of CE in PLWH confirms this data [117,118,119,120,121,122,123]: most of them had a full recovery after treatment. There are two cases of AE in seropositive patients [124,125], and a case of *Echinococcus vogeli*, causing polycystic echinococcosis [126].

Therefore, there seems to be a prevalence of CE among PLWH affected by echinococcosis, but HIV infection does not seem to affect the prognosis of echinococcosis. Further studies are needed to evaluate a possible relationship.

## 7. Brucellosis

Brucellosis is a bacterial infection usually transmitted by the consumption of raw meat or dairy products of infected animals. Other routes of infection are through the conjunctiva, respiratory tract or abraded skin. Symptoms of the infections are various and mostly nonspecific, such as fatigue and fever. Sweating with a strong odor is characteristic, but not always present. Hepatomegaly and splenomegaly can also occur. A frequent complication is osteoarticular compromise.

The most important species of Brucella for human health are *B. melitensis*, *B. abortus* and *B. suis*; *B. canis* is of less importance.

There is no strong association between brucellosis and HIV, and we did not find studies concerning brucellosis in PLWH in the Mediterranean regions. However, there are reports of brucellosis in specific settings that suggest a higher prevalence than expected. In a review conducted by Khademi et al. [127] on bacterial infections in hospitalized Iranian PLWH up to 2017, brucellosis was estimated to account for 26.3% of an overall rate of 48.6% of bacterial coinfections. Earlier in 2010, 90 seropositive patients were confronted with 100 controls in an Iranian cohort: the seroprevalence of brucellosis was significantly higher in PLWH than the controls, with a percentage of 73.3% vs. 30% [128]. In 2011, the prevalence of *Brucella* antibodies titer in 184 HIV-infected patients was assessed to be 6% (11 patients), with only three of them being symptomatic [129].

In Malawi, a sub-Saharan area, the prevalence of brucellosis among pregnant seropositive women was evaluated: 5 out of 201 (2.48%) had positive serology for *Brucella* and no symptoms, compatible with a previous infection [130].

Brucellosis seroprevalence was also assessed in Kenya, after the report of two patients with HIV and a serology positive for *Brucella*: among 100 patients, 65 of them being PLWH, 21 patients had IgG-serology for *Brucella*, 6 of them had IgM only, and 8 of them had both IgM and IgG, showing a rather significant prevalence of antibodies for *Brucella* [131].

About seven cases of brucellosis in PLWH are reported in the literature; most of them had complete recovery after diagnosis and opportune treatment [132,133,134,135,136,137].

In a small cohort of people of a case series reported by Moreno et al. [138], 12 people with HIV were diagnosed with brucellosis, mostly presenting the same clinical manifestation. In 11 of them, a common origin for the infection was identified.

HIV infection does not seem to affect *Brucella* prevalence, even though in endemic countries the incidence of brucellosis in PLWH is higher than expected; therefore, it is important to consider diagnosis of brucellosis in some specific geographic area and maintain attention on how it could affect immunocompromised people.

## 8. Rickettsiosis

*Rickettsia* is a group of obligate intracellular bacteria, widely distributed throughout the world. Rickettsiosis is divided into two main groups: the spotted fever group, transmitted by ticks or mites, and the typhus group, mainly transmitted by lice or fleas, and which comprehend *Rickettsia typhi* and *Rickettsia prowazekii*. *Rickettsia conorii* is one of the prevalent species in the Mediterranean area, causing the Mediterranean spotted fever, and it is usually associated with domestic pets.

*Rickettsia* infection usually leads to an increased vascular permeability and release of tumor necrosis factor alpha (TNF-alpha). Local rickettsiosis can be identified by a characteristic eschar with a necrotizing base. Disseminated rickettsiosis may lead to severe vasculitis, pneumonitis, meningoencephalitis and multiorgan failure. Nonspecific symptoms are fever, lymphadenopathy and diffused rash [139].

There is no strict association between rickettsiosis and HIV, as demonstrated by studies where seroprevalence for *Rickettsia* did not differ between the HIV group and the non-HIV group [140,141]. In a study in northeastern Spain, among 341 seropositive patients, 4.4% had positive serology for *R. felis* and 7.6% were seroreactive for *R. typhi*. Three patients had antibodies for both species [142].

There are three other cases, to our knowledge, of rickettsiosis in seropositive patients: a case of Mediterranean spotted fever, a case of rickettsialpox caused by *R. akari*, and a case of bilateral cilioretinal artery occlusion and uveitis in an HIV patient seroreactive for *Toxoplasma* and *Rickettsia* [142,143,144,145].

To date, *Rickettsia* does not seem to pose a threat for PLWH greater than that for the non-HIV-infected population, but it has to be considered among the possible diagnoses when clinically suggestive.

## 9. Borreliosis

A complex of spirochete bacteria, collectively known as *Borrelia burgdoferi*, is the cause of Lyme disease. Lyme disease is the most common vector borne disease in the United States and is also very prevalent in Europe. Borreliosis is transmitted by the bite of ticks that have been previously infected by biting other mammals. The characteristic skin lesion of Lyme disease is the erythema migrans, consisting of two concentric circular erythematous lesions. If the bacterium diffuses to other organs, it may cause alterations at the central nervous system (CNS), heart or joints [146,147].

Interestingly, a greater prevalence for IgM of *Borrelia* was demonstrated in the HIV group, with 227 patients, rather than the non-HIV group, in the same study where the seroprevalence for Rickettsia was tested [140]. Specifically, seroprevalence for IgM was 29.1%, and IgG, 4.8%.

In the literature, we found 13 cases of borreliosis in PLWH patients, most of them with a very similar presentation and course to the non-HIV-infected patients, and mostly presenting with cutaneous and/or neurological manifestations [148,149,150,151,152,153,154,155,156]. A woman undergoing treatment with ART had severe neurological sequelae after being treated for neuroborreliosis, and deambulation was especially compromised [157]. An atypical case was reported by Bratzke et al. [158], in which a patient with HIV was infected with *Borrelia* and developed a pseudolymphoma with unusual cytological features, lacking T-helper and Langerhans cells, but with an increased number of cytotoxic cells.

The correlation between *Borrelia* and HIV, or eventual complications of both infections, is unclear. In a study concerning depression and suicidal thoughts among patients with a history of Lyme disease in HIV and non-HIV patients, there was no significant difference in depression and development of suicidal thought between the two groups [159].

However, interestingly, the use of CXCL13 in the cerebrospinal fluid was suggested as a marker for neuroborreliosis, in comparison to other neurological infectious diseases. It was found that the protein levels decreased after treatment for borreliosis, but the marker levels were found elevated in 52% of the HIV untreated and neurologically asymptomatic patients, even considering that there was an overlap in the two groups [160]. Therefore, while a correlation between the two infectious diseases is still unclear, it seems that there are similar patterns mostly concerning neurological and psychiatric complications, such as depression, and biochemical markers.

## 10. Leishmaniasis

Leishmaniasis is a zoonosis caused by protozoan parasites, transmitted by the bite of infected female phlebotomine sandflies. Clinically, there are two forms of leishmaniasis: visceral leishmaniasis (VL) and cutaneous leishmaniasis (CL) [161]. *Leishmania infantum* causes both visceral leishmaniasis (VL) and cutaneous leishmaniasis (CL), and is endemic in the entire Mediterranean basin, while Leishmania major and *Leishmania tropica* causing CL are present only in countries of northern Africa and in Azerbaijan. Meanwhile, *L. donovani* sensu stricto has been found in certain areas of Cyprus and Turkey.

Leishmania and HIV coinfection was an emerging problem in the Mediterranean area during the 1990s, especially concerning Italy, Portugal, France and Spain. The introduction of ART has modified the history of this coinfection, achieving a 50–60% reduction in VL incidence [162]. A high incidence, especially in Spain, of Leishmania/HIV coinfection suggests that HIV infection might pose a risk for leishmaniasis and vice versa [163]. It has been shown that detectable peripheral blood parasitemia is more frequent in PLWH than in not-HIV-infected people [164]. Typical features of VL are fever, weight loss, hepatosplenomegaly and pancytopenia. Having common and non-specific features, acute visceral leishmaniasis may mask a primary HIV infection or, sometimes, reveal it [165]. Cutaneous involvement seems to be infrequent in HIV/Leishmania coinfected people. Meanwhile, sometimes, the inspection for skin lesions in PLWH, thought to be Kaposi Sarcoma, revealed cutaneous manifestation of Leishmania [166]. The severity of leishmaniasis in PLWH may be related to the shift towards a T helper 2 (Th2)-type specific response, instead of T helper 1, an amplification in NF-κB activation, and changes in CXCR4/CCR5 surface expression [167,168,169]. On the contrary, leishmaniasis seems to also affect and favor HIV progression to AIDS stage, accelerating the viral replication [170].

The interactions between the two infections constitute a major risk factor, not only for the severity of both diseases, but also for leishmaniasis reactivations, especially in people with a CD4+ count < 200 cell/uL [171].

Amphotericin B remains the first line option, with antimonials as the second line, even though lower efficacy of leishmaniasis treatment has been demonstrated in PLWH, with an impaired viro-immunological status [172]. The efficacy of secondary prophylaxis in PLWH is controversial, despite many studies having been conducted in the Mediterranean regions, mostly with amphotericin B lipid complex and liposomal amphotericin B [162].

## 11. Monkeypox

The emerging number of cases of monkeypox in European countries has brought renewed attention to this disease, which was originally isolated in humans in 1970. Monkeypox is a zoonotic disease caused by an orthopoxvirus, resulting in a smallpox-like disease in humans. Since its first isolation, monkeypox has become endemic in the Democratic Republic of Congo (DRC) and other African regions. In addition, the median age has varied over the years, from young children to young adults, probably due to the cessation of smallpox vaccination, which probably also provided some sort of protection against monkeypox.

Initial symptoms include fever, fatigue and headache. Generalized lymphadenopathy, mostly in maxillary, cervical and inguinal regions is typical, along with a rash, first macular, then papular, then vesicular and pustular, which appears first on the head region and then diffuses in other parts of the body with centrifugal distribution [173].

Among cases with known HIV status in EU countries and up to July 2022, 40% (364/917) were HIV-infected. A specific case of monkeypox in a HIV patient has been reported in the literature: a male in his 30s, viro-suppressed and with a CD4+ count above 700 cells/uL, had genital rash, with no other initial symptoms. He then developed malaise and fever and the rash diffused on the rest of the body. He was admitted to hospital and started on antibiotics. The man’s condition improved, and he was discharged [174]. Even if the infection did not seem to have any sequelae, the risk of uncontrolled HIV infection may be a major risk factor for the increasing number of monkeypox cases. Moreover, in the case described, the infection was sexually transmitted, so unsafe sexual behavior has to be considered a major concern not only for HIV and other sexually transmitted diseases (STD), but for monkeypox too.

## 12. Conclusions

It is known that HIV infection in the absence of ART causes immunodeficiency that may lead to opportunistic infections from neglected pathogens, which normally, in an immunocompetent person, do not cause severe disease. In some cases, as in the case of *Salmonella*, the infection was recognized as an AIDS-defining illness. In other cases, there does not seem to be a strong correlation with HIV. The introduction of ART has surely changed the odds, and pathogens such as *Campylobacter* are no longer more virulent than they are in immunocompetent people. However, as observed, in PLWH under ART, there are alterations in the microbiome for these pathogens or overlapping symptoms, as in the case of neuroborreliosis. Thus, even if the epidemiology of the most common zoonoses in the Mediterranean do not seem to have a strict relationship with HIV, they have to be kept in mind when formulating a diagnosis, as well as the possible consequences. Further studies are needed to explore a possible interaction between these important infectious diseases.

## Figures and Tables

**Table 1:** Epidemiology of some of the most common zoonoses in European countries as reported by the ECDC bulletin, referring to 2020 (note: monkeypox epidemiology updated to 7 July 2022).

Zoonoses Epidemiology in EU in 2020	Cases	Hospitalizations	Deaths
Campylobacteriosis	120,946	8605	45
Salmonellosis	52,702	6149	57
Listeriosis	1876	780	167
Echinococcosis	488	44	0
Brucellosis	128	36	2
Rickettsiosis	-	-	-
Borreliosis	-	-	-
Leishmaniasis	-	-	-
Monkeypox *	4908 *	-	0 *

Epidemiology of most common zoonoses; * Refers to July 2022 epidemiology.

## Data Availability

Not applicable.
